# Empathy, emotional intelligence, and communication in Nursing: The moderating effect of the organizational factors*

**DOI:** 10.1590/1518-8345.3286.3333

**Published:** 2020-08-12

**Authors:** María del Carmen Giménez-Espert, Elena Castellano-Rioja, Vicente Javier Prado-Gascó

**Affiliations:** 1 University of Valencia, Valencia, Spain.; 2 Catholic University of Valencia “San Vicente Martir”, Valencia, Spain.

**Keywords:** Communication, Contracts, Emotional Intelligence, Empathy, Nursing Service, Hospital, Comunicação, Contratos, Inteligência Emocional, Empatia, Serviço Hospitalar de Enfermagem, Comunicación, Contratos, Inteligencia Emocional, Empatía, Servicio de Enfermería en Hospital

## Abstract

**Objective:**

to evaluate the relation and the moderating effect of the organizational factors on the attitudes towards communication, empathy, and emotional intelligence in the nurses.

**Method:**

a cross-sectional study was conducted with a convenience sample of 268 nurses from Valencia, Spain. The attitudes towards communication were evaluated by means of the specifically designed instrument, those towards empathy with the Jefferson’s Scale of Empathy for Nursing Students, and those towards emotional intelligence by means of the Trait Meta-Mood Scale, consisting of 24 items. The effect of the studied variables was assessed by means of ANOVA, multiple linear regression models were applied, and the moderating effect was analyzed using PROCESS.

**Results:**

there are statistically significant differences based on the type on contract (permanent); and statistically significant differences were found in the cognitive dimension of the attitudes towards communication. Regarding the regression models, the perspective taking dimension of empathy was the main predictive variable tn the dimensions of the attitudes towards communication. Finally, a moderating effect of the type of contract was evidenced in the effect of emotional reparation over the cognitive dimension of the attitudes towards communication.

**Conclusion:**

the organizational factors exert an influence on the attitudes towards communication, empathy, and emotional intelligence.

## Abstract (pt)

**Objetivo:**

avaliar a relação e o efeito moderador dos fatores organizacionais nas atitudes dos enfermeiros em relação à comunicação, empatia e inteligência emocional.

**Método:**

estudo transversal em amostra de conveniência de 268 enfermeiros de Valência, Espanha. As atitudes em relação à comunicação foram avaliadas usando o instrumento desenvolvido para esse fim, a empatia com a *Jefferson Scale of Empathy for Nursing Student* e a inteligência emocional usando a *Trait Meta-Mood Scale* de 24 itens. O efeito das variáveis estudadas foi avaliado por meio de ANOVA, modelos de regressão linear múltipla foram realizados e o efeito moderador foi analisado por PROCESS.

**Resultados:**

existem diferenças estatisticamente significativas dependendo do tipo de contrato (permanente); e diferenças estatisticamente significantes foram encontradas na dimensão cognitiva das atitudes em relação à comunicação. Em relação aos modelos de regressão, a dimensão tomada de perspectiva da empatia foi a principal variável preditora nas dimensões das atitudes em relação à comunicação. Finalmente, um efeito moderador do tipo de contrato foi demonstrado no efeito do reparo emocional sobre a dimensão cognitiva das atitudes em relação à comunicação.

**Conclusão:**

fatores organizacionais influenciam atitudes em relação à comunicação, empatia e inteligência emocional.

## Abstract (es)

**Objetivo:**

evaluar la relación y el efecto moderador de los factores organizacionales sobre las actitudes hacia la comunicación, empatía e inteligencia emocional de los enfermeros.

**Método:**

se realizó un estudio transversal en una muestra por conveniencia de 268 enfermeros de Valencia, España. Las actitudes hacia la comunicación se evaluaron mediante el instrumento diseñado para tal fin: la empatía con la *Jefferson Scale of Empathy for Nursing Student* y la inteligencia emocional mediante la *Trait Meta-Mood Scale* de 24 ítems. El efecto de las variables estudiadas se evaluó mediante ANOVA, se realizaron modelos de regresión lineal múltiple y el efecto moderador se analizó mediante PROCESS.

**Resultados:**

existen diferencias estadísticamente significativas en función del tipo de contrato (permanente); se encontraron diferencias estadísticamente significativas en la dimensión cognitiva de las actitudes hacia la comunicación. Con respecto a los modelos de regresión, la dimensión toma de perspectiva de la empatía fue la principal variable predictora en las dimensiones de las actitudes hacia la comunicación. Finalmente, se evidenció un efecto moderador del tipo de contrato en el efecto de la reparación emocional sobre la dimensión cognitiva de las actitudes hacia la comunicación.

**Conclusión:**

los factores organizacionales influyen en las actitudes hacia la comunicación, empatía e inteligencia emocional.

## Introduction

Communication is a complex, multi-dimensional, and dynamic process, which refers to developing the means of interaction in an effective and efficient way^(^ 1 ^)^; it constitutes itself as one of the most important indicators to assess the quality of Nursing care^(^ 2 ^-^ 3 ^)^. It is for this reason that the Joint Commission International (JCI), as a governmental organization for the global accreditation of the best practices related to quality and to patients’ safety, identifies inadequate communication among the most common causes of adverse events. Effective communication helps to improve the care actions^(^ 4 ^)^ and the patients’ health results^(^ 1 ^,^ 5 ^)^, as well as it contributes to the team’s satisfaction and to organizational effectiveness^(^ 6 ^)^. However, despite its importance, studies have barely been developed on the nurses’ communication with the patients in the hospital general context^(^ 6 ^)^, in clear opposition to the case of Oncology and in long-term care settings^(^ 7 ^)^, ignoring other situations or realities.

Studying communication as a human conduct inevitably lies on evaluating the attitudes towards such conduct^(^ 8 ^)^. Additionally, in developing communication, Emotional Intelligence (EI from now on)^(^ 9 ^)^ and empathy^(^ 10 ^)^ play a fundamental role. In this sense, there are studies on the relations of the attitudes towards communication, EI, and empathy^(^ 11 ^-^ 12 ^)^. These variables can be influenced by intrinsic and extrinsic factors, like the type of service where the nurses work (general hospitalization or special services) and the type of contract (permanent or temporary)^(^ 13 ^-^ 14 ^)^. Work uncertainty^(^ 15 ^)^, related to the type of contract, as well as the stress level in the service (general hospitalization or special services), can hinder communication; but EI and empathy facilitate communication even in stressful moments^(^ 16 ^)^, hence their study is considered fundamental. However, despite their importance, we have not found in the literature any study which analyses the relation and moderating effect of these extrinsic factors (type of service and the nurses’ contracts) on the attitudes towards communication, empathy, and EI in the nurses. It is for this reason that the main objective of our paper is to analyze the impact of these factors.

## Method

A cross-sectional study was designed, with a convenience sample consisting of 286 nurses from 3 hospitals in Valencia, Spain. The calculation for the sample size was performed according to the population of 2,000 nurses who worked in the participating hospitals, taking into account a 95% confidence interval and an alpha error of 5.9%, a sample size of 243 nurses being necessary. Eventually, the study’s sample size was 286 nurses. The inclusion criteria were the following: being an active working nurse in the selected hospitals and having expressed their informed and voluntary consent to participate. The exclusion criteria were the following: being on vacation, time-off, and leave periods during the three weeks of data collection. In the active working situation of the nurses, the type of contract (temporary and permanent) was considered. In Spain, a permanent contract offers more stability because the nurses have passed a selective process, thus obtaining an appointment to perform their duties permanently. In addition, the nurses who hold this condition can participate in internal promotion and staff mobility processes. In the case of temporary contracts, the nurse is appointed by the health services due to need or urgency, or for developing programs of a temporary nature; the job, therefore, is performed for a limited period of time^(^ 17 ^)^.

Data collection was conducted by means of a self-applied instrument. The participants had to answer based on a Likert format with five options, ranging from 1 = Totally disagree to 5 = Totally agree. The instrument contained the following scales:

- Sociodemographic data: work center, service, gender, age, and working situation.
- A questionnaire on the nurses’ attitudes towards communication (ACO)^(^ 18 ^-^ 19 ^)^. It consists of 25 items grouped into three dimensions: affective, cognitive, and conative, to assess the attitudes towards communication. This instrument has shown adequate psychometric properties: Satorra-Bentler (S-B χ2); Degrees of Freedom (DoF) S-B χ2(DoF)=525.09 (272); χ2(DoF)=4.90*;* Root Mean Square Error of Approximation (RMSEA); Confidence Interval (CI); RMSEA (CI)=0.045 (0.037-0.057); Comparative Fit Index (CFI), CFI=0.91, Non-Normalized Fit Index (NNFI), NNFI=0.90, IFI=0.91; Affective: Cronbach’s alpha confidence interval (CIα)=0.95 (0.94-0.96); Compound Reliability Coefficient (CRC), CRC=0.95; Average Variance Extracted (AVE), AVE=0.60; Conative: CIα=0.92 (0.90-0.93), CRC=0.91, AVE=0.53; Cognitive: CIα=0.85 (0.82-0.87), CRC=0.85; AVE=0.58^(^ 18 ^)^.
- The Jefferson’s Scale of Empathy for Nursing Students adapted from Jefferson’s Scale of Physician Empathy (JSPE)^(^ 20 ^)^ was translated by the research team. Jefferson’s medical empathy scale was adapted from Nursing students to the nurses; in its original version^(^ 20 ^)^, it had 19 items (JSNE) grouped into three factors to assess empathy. It presents adequate psychometric properties: Satorra-Bentler (S-B χ2); Degrees of Freedom (DoF); S-B χ2(DoF)=174.74 (87); χ2(DoF)=3.82; RMSEA (CI)=0.047 (0.037-0.057); CFI=0.92, NNFI=0.90, IFI=0.91; Perspective taking: Cronbach’s alpha confidence interval (ICα)=0.87 (0.85-0.89); CRC=0.88 AVE=0.47; Compassionate care: ICα=0.78 (0.75-0.81); CRC=0.78; AVE=0.48; Thinking as the patient: ICα=0.76 (0.71-0.80);CRC=0.76; AVE=0.61^(^ 21 ^)^.
- The Trait Meta-Mood Scale (TMMS24) is a scale consisting of 24 items grouped into three dimensions, the Spanish version having been adapted by Fernández-Berrocal and to the Nursing context^(^ 22 ^)^ to assess EI. It presents adequate psychometric properties in the Nursing population: Satorra-Bentler (S-B χ2); Degrees of Freedom (DoF); S-B χ2(DoF)=370.20 (149); χ2(DoF)=3.58; RMSEA (IC)=0.057 (0.050-0.65); CFI=0.91; NNFI=0.90; IFI=0.91; Emotional care: Cronbach’s alpha confidence interval (CIα)=0.80 (0.77-0.83), CRC=0.80; AVE=0.45; Emotional clarity: CIα=0.87 (0.85-0.89), CRC=0.87; AVE=0.46; Emotional reparation: CIα=0.85 (0.82-0.87); CRC=0.85; AVE=0.49^(^ 21 ^)(^ 23 ^)^.

Firstly, the differences in the study variables were analyzed according to the type of contract and service by means of the Student’s t test. Multiple models of hierarchical linear regression were calculated considering the type of contract (temporary and permanent) and service (general hospitalization and special services). Finally, the moderating effect was calculated of the type of contract and service on the effect of JSNE and of TMMS24 over ACO by means of 18 different models, considering each of the independent variables (dimensions from JSNE and from TMMS24) over the criteria variables (ACO dimensions). These analyses were performed using the PROCESS^(^ 24 ^)^ macro software installed in the Statistical Package for the Social Sciences (SPSS), version 23, designed to test moderation by means of the direct evaluation of the importance of the indirect effect of the independent variable on the dependent variable through two moderators: Working situation (M) and Type of service (W). The moderating effect (with n = 5,000 bootstrap samples) is demonstrated when the corrected confidence interval (95%) of the indirect effect does not include zero^(^ 24 ^)^. All these analyses were performed using the Statistical Package for the Social Sciences (SPSS), version 23.

The study was approved by the Research Ethics Committee of the University of Valencia (H143203222268924) and by the Committees of Ethics in Clinical Research (*Comités de* Ética *de la Investigación Clínica*, CEIC) of the selected hospitals. All the individuals who agreed to participate had previously received detailed information about the study, as well as on the confidentiality of the data provided.

## Results

A total of 268 nurses answered the questionnaires and 12 were excluded due to not having completed them properly; consequently, 286 questionnaires were analyzed. Regarding the sociodemographic characteristics of the participants, their age ranged from 23 to 63 years old, with a mean of 45.25 (Standard Deviation=10.97). In terms of gender distribution, 74.1% (213) are women and 25.5% (73) are men. Regarding the working situation of the participants, 47.9% (137) are temporary workers, whereas 52.1% (149) have a permanent job position. As regards the type of service, 56.6% (162) works in hospitalization units and 43.4% (124) in special services.

According to the differences in ACO, JSNE, and TMMS24 based on the type of contract and service, statistically significant differences (p≤.05) were only found in the cognitive dimension (t=-2.41; p=.017; η2=.02) of the ACO scale considering the type of contract. The Nursing staff with a permanent working situation presented a slightly higher score (Mean=4.52, Standard Deviation=0.76) than the temporary personnel (Mean=4.26, Standard Deviation=0.98) (Table 1).

**Table 1:** Dimensions of the attitudes towards communication, Jefferson's Scale of Empathy for Nurses and Trait Scale of Meta-knowledge on Emotional States (or Trait Meta-Mood Scale) according to the type of contract. Valencia, Spain, 2016

	Dimensions	Temporary contract		Permanent contract		t test*	*p* value^†^	Effect^‡^
		Mean	Standard Deviation	Mean	Standard Deviation			
Attitudes towards communication	Affective	1.71	0.96	1.57	0.82	1.25	0.21	^§^
	Conative	4.02	0.86	4.18	0.79	-1.56	0.12	^§^
	Cognitive	4.26	0.98	4.52	0.76	-2.41	0.017^‖^	0.02
Jefferson's empathy scale	Perspective taking	4.49	0.55	4.47	0.62	0.35	0.73	^§^
	Compassionate care	1.98	0.90	1.95	0.89	0.21	0.83	^§^
	Thinking as the patient	2.03	1.04	2.08	1.06	-0.38	0.70	^§^
Trait Meta Mood Scale	Emotional care	3.46	0.74	3.57	0.71	-1.21	0.23	^§^
	Emotional clarity	3.76	0.61	3.74	0.73	0.30	0.77	^§^
	Emotional reparation	3.77	0.74	3.77	0.76	0.05	0.96	^§^

* t-test;

† *p*value resulting from the Levene test;

‡ Effect;

§ It was not calculated because there were no significant differences; p≤0.01;

‖ p≤0.05

On the other hand, when considering the type of service, no significant differences were observed between general hospitalization and special services (Table 2).

**Table 2:** Dimensions of the attitudes towards communication, Jefferson's Scale of Empathy for Nurses and Trait Scale of Meta-knowledge on Emotional States (or Trait Meta-Mood Scale) according to the type of service. Valencia, Spain, 2016

	Dimensions	General hospitalization		Special service		t test*	*p* value^†^	Effect^‡^
		Mean	Standard Deviation	Mean	Standard Deviation			
Attitudes towards communication	Affective	1.59	0.88	1.69	0.91	-.87	0.39	^§^
	Conative	4.12	0.80	4.08	0.87	0.31	0.76	^§^
	Cognitive	4.43	0.89	4.35	0.88	.75	0.46	^§^
Jefferson's empathy scale	Perspective taking	4.48	0.58	4.49	0.60	-.14	0.89	^§^
	Compassionate care	2.05	0.92	1.85	0.85	1.83	0.07	^§^
	Thinking as the patient	2.05	1.09	2.06	1.00	-0.05	0.96	^§^
Trait Meta Mood Scale	Emotional care	3.57	0.75	3.45	0.68	1.25	0.21	^§^
	Emotional clarity	3.79	0.67	3.69	0.67	1.22	0.22	^§^
	Emotional reparation	3.75	0.79	3.80	0.70	-0.54	0.59	^§^

* t-test;

† *p*value resulting from the Levene test;

‡ Effect;

§ It was not calculated because there were no significant differences; p≤0.01; p≤0.05

Subsequently, the multiple linear regression models were analyzed based on the type of contract and service. The predictive value of EI and of empathy on the dimensions of the ACO instrument considering the type of contract (temporary or permanent) and of service (general hospitalization or special service) was analyzed by means of hierarchical regression models. In all cases, in the first step the empathy dimensions (JSNE) were included and, in the second, those of EI (TMMS24).

Considering the type of contract (Table 3), in the case of those individuals with a temporary contract, the variables taken into account were capable of explaining 7% of the affective dimension (R^2^_adjusted_=0.07, *p*≤0.05), 11% of the cognitive dimension (R^2^_adjusted_=0.11, *p*≤0.01), and 17% of the conative dimension (R^2^_adjusted_=0.17, *p*≤0.01). On the other hand, considering those individuals with a permanent contract, the variables taken into account explained 14% of the affective dimension (R^2^_adjusted_=0.14, *p*≤0.01), 16% of the cognitive dimension (R^2^_adjusted_=0.16, *p*≤0.01), and 29% of the conative dimension (R^2^_adjusted_=0.29, *p*≤0.01).

**Table 3:** Hierarchical regression analysis for the dimensions from JSNE and from TMMS24 over the dimensions from ACO (affective, cognitive, and conative) based on the type of contract. Valencia, Spain, 2016

Variable	Affective		Cognitive		Conative	
Predictors	∆R^2^	Β	∆R^2^	β	∆R^2^	Β
Step 1 Temporary	0.06		0.13*		0.17^†^	
Perspective taking		-0.19		0.32^†^		0.40^†^
Compassionate care		0.07		-0.09		0.07
Thinking as the patient		0.06		0.01		-0.13
Step 2 Temporary	0.06		0.03		0.05	
Perspective taking		-0.22		0.28^‡^		0.30*
Compassionate care		0.04		-0.09		0.06
Thinking as the patient		0.07		0.01		-0.12
Emotional care		0.08		-0.03		-0.06
Emotional clarity		-0.23^‡^		0.18		0.22^‡^
Emotional reparation		0.17		-0.01		0.07
Total R^2^_adjusted_	0.07^‡^		0.11^†^		0.17^†^	
Step 1 Permanent	0.14^†^		0.17^†^		0.29^†^	
Perspective taking		0.14^†^		0.38^†^		0.54^†^
Compassionate care		0.11		0.01		0.22^‡^
Thinking as the patient		0.08		-0.08		-0.17
Step 2 Permanent	0.05		0.03	-	0.04	
Perspective taking		0.15*		0.41^†^		0.54^†^
Compassionate care		0.10		-0.00		0.22^‡^
Thinking as the patient		0.08		-0.07		-0.17
Emotional care		0.12		-0.12		-0.15
Emotional clarity		0.13^‡^		0.20		0.00
Emotional reparation		0.13		-0.18		0.15
Total R^2^_adjusted_	0.14^†^		0.16^†^		0.29^†^	

* p≤.01;

† p≤.001;

‡ p≤.05

Specifically, in the case of the workers with a temporary contract, only the perspective taking dimension from JSNE for the prediction of the conative (β = 0.30; p≤0.01) and cognitive (β *= 0.28; p*≤0.05) dimensions, and the emotional clarity dimension from TMMS24 for the prediction of the affective (β = -0.23; p≤0.05) and conative (β = 0.22; p≤0.05) dimensions turned out to be significant. Regarding the workers with a permanent contract, it was observed that perspective taking in JSNE positively and significantly predicted the three dimensions from ACO: affective, cognitive, and conative (β = 0.15; *p*≤0.01, β =0.41; *p*≤0.001, β= 0.54; *p*≤0.001). The emotional clarity dimension from TMMS24 positively and significantly predicted the affective dimension from ACO (β = 0.13; p≤0.05), and the compassionate care dimension positively and significantly predicted the conative dimension from ACO (β = 0.22; p≤0.05) (Table 3).

In the type of service model (Table 4), in the case of the general hospitalization workers, the variables taken into account were capable of explaining 15% of the affective dimension (R^2^_adjusted_=0.15, *p*≤0.01), 17% of the cognitive dimension (R^2^_adjusted_=0.17, *p*≤0.01), and 25% of the conative dimension (R^2^_adjusted_=0.17, *p*≤0.01). On the other hand, regarding the nurses who work in special services, the variables taken into account explained 6% of the affective dimension (R^2^_adjusted_=0.06, *p*≤0.05), 8% of the cognitive dimension (R^2^_adjusted_=0.08, *p*≤0.05), and 19% of the conative dimension (R^2^_adjusted_=0.19, *p*≤0.01).

**Table 4:** Hierarchical regression analysis for the dimensions from JSNE and from TMMS24 over the dimensions from ACO (affective, cognitive, and conative) based on the type of service. Valencia, Spain, 2016

Variable	Affective		Cognitive		Conative	
Predictors	∆R^2^	β	∆R^2^	β	∆R^2^	β
Step 1 General hospitalization	0.09		0.16*		0.23*	
Perspective taking		-0.27^†^		0.36*		0.48*
Compassionate care		0.03		-0.05		0.23^‡^
Thinking as the patient		0.05		-0.03		-0.18^‡^
Step 2 General hospitalization	0.01^†^		0.05		0.05^‡^	
Perspective taking		-0.25^‡^		0.36*		0.40*
Compassionate care		0.01		-0.03		0.21^‡^
Thinking as the patient		0.03		-0.03		-0.15
Emotional care		0.17		-0.11		-0.16
Emotional clarity		-0.35*		0.24^‡^		0.24^‡^
Emotional reparation		0.21^‡^		-0.18		0.03
Total R^2^_adjusted_	0.15*		0.17*		0.25*	
Step 1 Special services	0.11^‡^		0.13^‡^		0.22*	
Perspective taking		0.14*		0.31^†^		0.43*
Compassionate care		0.11		-0.07		0.02^‡^
Thinking as the patient		0.08		-0.01		-0.11^‡^
Step 2 Special services	0.02		0.01		0.03	
Perspective taking		0.15^†^		0.26^‡^		0.35^†^
Compassionate care		0.10		-0.07		0.01
Thinking as the patient		0.08		-0.02		-0.11
Emotional care		0.12		0.03		-0.02
Emotional clarity		0.13		-0.00		-0.06
Emotional reparation		0.13		0.12		0.20
Total R^2^_adjusted_	0.06^‡^		0.08^‡^		0.19*	

* p≤.001;

† p≤.01;

‡ p≤.05

Specifically in the case of the general hospitalization services, the perspective taking dimension from JSNE positively and significantly predicted the cognitive (β = 0.36; p≤0.001) and conative (β = 0.40; p≤0.001) dimensions from ACO and negatively predicted the affective dimension from ACO (β = -0.25; p≤0.05). The emotional clarity dimension fromTMMS24 negatively and significantly predicted the affective dimension from ACO (β = -0.35; p≤0.001) and positively predicted the cognitive (β = 0.24; p≤0.05) and conative (β = 0.24; p≤0.05) dimensions. In the case of the special services, only the perspective taking dimension from JSNE positively and significantly predicted the three dimensions from ACO: affective, cognitive, and conative (β = 0.15; *p*≤0.01, β = 0.26; *p*≤0.05, β = 0.35; *p*≤0.01) (Table 4).

Finally, based on the analysis of the type of contract and service over the effect of empathy and EI on the attitudes towards communication, a moderating effect of the working situation on the effect of emotional reparation over the cognitive dimension of the ACO instrument was only observed, since it is demonstrated when the corrected confidence interval (95%) of the indirect effect does not include zero^(^ 23 ^)^ (Lower confidence interval = 0.0440; Higher confidence interval = 0.6152). Specifically, emotional reparation seems to be a better predictor in the case of the permanent contracts (β = 0.21; *p=0.03*) than in the temporary ones (β = 0.14; *p=0.10*). (Effect_permanent_=0.22; SE_permanent_=0.10; t_permanent_=2.15, *p*_permanent_=0.03; Effect_temporary_=0.14; SE_temporary_=0.10; t_temporary_=1.43 *p*_permanent_=0.15).

## Discussion 

The communication, empathy and EI skills had a direct repercussion on the quality of care^(^ 2 ^-^ 3 ^)^. It was important to consider external factors which were more related to the organizational aspects, like the type of contract and services, which could have exerted an influence on those skills^(^ 13 ^-^ 14 ^)^. However, there are still no studies which analyze these relations; so the objective of this research was to know how the type of contract and service affected the attitudes towards communication, empathy, and EI in the nurses.

The Nursing literature presents studies on the influence of the organizational aspects on their work satisfaction and on the nurses’ intention to leave their profession, together with the appearance of negative feelings of impotence and uselessness^(^ 25 ^-^ 26 ^)^. Other studies assess quality of life in the work of the Nursing personnel based on the type of contract and on the type of institution where they work^(^ 6 ^)^, as well as the psychosocial factors and the mental burden of the nurses related to the care unit where they work^(^ 27 ^)^. These studies show the need to foster work environments where work security favors satisfaction, professional development, and personal well-being. In this research, apart from approaching the aspects related to the extrinsic factors (type of contract and service), intrinsic factors like communication, empathy, and EI are also considered.

The results of our study show that there only seems to be statistically significant differences in the case of the cognitive dimension from the ACO scale considering the type of contract; those professionals with a permanent contract seem to present slightly higher levels than those with a temporary contract.

Likewise, the empathy and EI dimensions are better predictors of the attitudes towards communication in the case of individuals with a permanent contract than in those with a temporary contract. Similarly, those variables were also better predictors in the case of the general hospitalization services compared to the special services. In general, the variable which best seems to predict the attitudes towards communication is perspective taking (JSNE), followed by emotional clarity. Finally, a moderating effect of the working situation on the effect of emotional reparation was observed over the cognitive dimension of the ACO instrument; in this sense, emotional reparation seems to be a better predictor in the case of the permanent contracts.

These results might indicate that work uncertainty^(^ 15 ^)^, related to the type of contract, as well as the stress level in the service (general hospitalization or special services), can hinder communication. In this study we found studies which relate work insecurity and stress level in the service as negative factors for providing quality Nursing care and for the nurses’ well-being^(^ 6 ^,^ 25 ^-^ 26 ^)^. The results of this research also showed that empathy and EI constitute themselves as protection elements in these situations^(^ 16 ^)^.

Despite the contributions of this research, it does have limitations: the sampling procedures are not probabilistic, as well as the geographical context, since the sample consists only of nurses working in hospitals of Valencia, Spain. It is due to these aspects that the results are not representative of all the Nursing professionals, which hinders the generalization of the results found. In the future it is proposed to conduct a stratified probabilistic sampling, considering different geographical areas, thus improving data generalization.

## Conclusion

Nurses with a permanent contract seem to present slightly higher levels in the cognitive dimension from the ACO scale, which evidences the importance given to the communication with the patients and/or families. In the case of the permanent contracts and of the general hospitalization services, the empathy and EI dimensions are better predictors of the attitudes towards communication.

Our study shows that the type of contract and service exert an influence on the attitudes towards communication, empathy, and EI, with the consequent possibility of arising as risk or protection factors. Thus, improving the working conditions of the Nursing professionals will translate into the improvement of their attitudes towards communication and, therefore, of the quality of the Nursing care.
